# Antiretroviral therapy adherence strategies used by patients of a large HIV clinic in Lesotho

**DOI:** 10.1186/s41043-015-0026-9

**Published:** 2015-08-06

**Authors:** Johanna Maria Axelsson, Sofie Hallager, Toke S. Barfod

**Affiliations:** 1 Department of Medicine, Copenhagen University Hospital Glostrup, Glostrup, Denmark; 2 Department of Infectious Diseases, Hvidovre University Hospital, Hvidovre, Denmark; 3 Department of Medicine, Roskilde University Hospital, Roskilde, Denmark

**Keywords:** Adherence, Antiretroviral Therapy, Barriers, Facilitators, HIV/AIDS, Lesotho, Sub-Saharan Africa

## Abstract

A high degree of adherence to antiretroviral therapy (ART) in patients infected with *human immunodeficiency virus (*HIV) is necessary for long term treatment effects. This study explores the role of timing of ART intake, the information patients received from health workers, local adherence patterns, barriers to and facilitators of ART among 28 HIV-positive adults at the Senkatana HIV Clinic in Maseru, Lesotho. This qualitative, semi-structured interview study was carried out during February and March of 2011 and responses were analyzed inspired by the Grounded Theory method. Results were then compared and discussed between the authors and the main themes that emerged were categorized. The majority of the respondents reported having missed one or more doses of medicine in the past and it was a widespread belief among patients that they were required to skip the dose of ART if they were “late”. The main barriers to adherence were interruptions of daily routines or leaving the house without sufficient medicine. The use of mobile phone alarms, phone clocks and support from family and friends were major facilitators of adherence. None of the patients reported to have been counseled on family support or the use of mobile phones as helpful methods in maintaining or improving adherence to ART. Being on-time with ART was emphasized during counseling by health workers. In conclusion, patients should be advised to take the dose as soon as they remember instead of skipping the dose completely when they are late. Mobile phones and family support could be subjects to focus on during future counseling particularly with the growing numbers of mobile phones in Africa and the current focus on telemedicine.

## Background

The prevalence of infections with the human immunodeficiency virus (HIV) in Lesotho among adults was estimated to be 23.1 %, the second highest HIV prevalence in the world, with an estimated 15,000 AIDS related deaths per year in 2012 [1]. Fifty-nine percent of eligible patients in Lesotho were estimated to receive antiretroviral therapy (ART) in 2012 [1].

The introduction of ART has changed HIV from a deadly to a chronic infection for people living with HIV (PLWH). Research has shown that there is a relationship between the level of adherence to ART and disease progression [2] and mortality [3]. Current ART can supress viral load at moderate adherence levels (70–90 % of prescribed doses taken over a given time), to receive the best outcomes, however, ART should be taken correctly as prescribed [4].

Non-adherence increases the risk of development of resistance [5, 6] and since the cost of second-line therapy is considerably higher than first-line therapy, high levels of adherence is important for cost control and improved survival especially in resource-limited countries [7].

Self-reported adherence is the most widely used method to estimate adherence although it is prone to overestimate the level of adherence [8]. In a meta-analysis of 27 Sub-Saharan African studies conducted prior to 2006, 23 % of the patients receiving ART had sub-optimal levels of adherence, defined by local thresholds [9]. Sub-optimal adherence is caused by different barriers to treatment adherence and frequently reported barriers in Sub-Saharan Africa include: transport costs [10–12]; lack of social support (financial and emotional) [13, 14]; service-related factors [10–14]; pill burden and side-effects [11–14]; poor knowledge about HIV; stigma [10–12]; non-disclosure of HIV status[10, 12, 14]; and a lack of food [12, 14]. Furthermore, adherence tend to decline over time [15–17] and a major challenge in resource poor settings is yet to provide ART to all eligible patients [18, 19].

To our knowledge no previous studies have been conducted exploring the information obtained during counselling on the timing of ART intake. In more affluent countries, it is suggested that HIV-positive patients are advised to take their daily ART in conjuction with daily routines, such as eating dinner or brushing their teeth [20]. However, it is our personal experience from clinical work in Lesotho and Botswana and from discussions with Sub-Saharan African colleagues that patients are often told to take their ART *“on time”*. “*On time*” in this setting indicates to take the daily doses of ART at the exact same time every day. This practice may cause patients who discover that they are late, to completely skip the dose. Thus, the aims of this study was to explore adherence patterns, describe barriers to and facilitators of adherence to ART, and to investigate the role of “timing of doses” and information received at the clinic about “timing of doses”.

## Methods

### Participants, procedures and settings

The Senkatana HIV clinic had two physicians and three nurse practitioners employed. The number of patients visiting the clinic regularly was estimated to be within the range of 6000–9000 patients. The clinic in Maseru were in 2011 one of over 30 publicly funded clinics in the city. The clinic provided counseling, ART and a few other drugs for free but not travels expenses or nutritional supplements. Prior to initiation of ART, patients were counseled by adherence counselors (nurse practitioners). Patients were given medication for one month at a time. For adherence control, patients provided their remaining medication and an adherence calendar (“mokoko”) every month to the pharmacists. If patients had been non-adherent, they were told by the pharmacists to take their medicine. The pharmacists referred patients to a physician for adherence counselling if necessary. Patients were offered brief counselling during a follow-up with a physician every 3 months.

PLWH at the Senkatana HIV Clinic in Maseru, Lesotho, were purposefully selected by non-random sampling as they were awaiting their monthly clinic visit, during February and March of 2011. Patients were approached by the author, T.B., and two local research assistants. Our sampling goal was to obtain a variety among patients in terms of gender and age. Eligible patients were 18 years of age and older, HIV-positive and receiving ART. They were orally informed and gave oral consent to participation in the study. No financial compensation was given. It was emphasized that participation was entirely voluntary, all interviews were confidential and that whether the patients participated or not would not affect their treatment at the clinic. A total of 30 patients were approached for participation, however, two patients declined to participate. Both gave as reason that they wished to be financially compensated. The interview-guide consisted of 17 semi-structured questions and 14 demographic, structured questions. The author T.B. created the questionnaire based on his experience with qualitative research conducted in Denmark among PLWH and on clinical experience in low-and middle income countries. Originally written in English, the interview-guide was translated into the local language Sesotho and double checked by three independent translators. The set of questions were constructed to be comprehensive with respect to the relevant themes of adherence in an everyday setting. Each individual interview followed its own path and depended on the patient’s answers. The casual form of the interviews gave the interviewer the opportunity to ask the informant to further specify certain answers. Questions regarding adherence were focused on facilitators and barriers to adherence, such as what made it difficult or easy to take the daily ART. Patients were asked about demographics including age, gender, and language, names of medicine, duration on ART, ART regimen, education and monthly household income. Patients were asked whether they had ever missed a dose of ART and how many days they had missed ART in the past month and year. Fifteen interviews were carried out by the senior author, T.B. A local translator was used in cases where the patient did not speak English. The remaining 13 interviews were carried out by two local research assistants fluent in both Sesotho and English and carefully instructed in interviewing techniques. The response to each question was written down in English during the interview, the phrasing as close to the spoken words as possible, in a slightly abbreviated form. All interviews were discussed with the author, T.B., on the same day to clarify any misunderstandings and ambiguous answers. The research protocol was approved by the Director General of Health Services in Maseru, Lesotho.

### Data analysis and management

All handwritten answers were manually converted into digital forms using Microsoft Words 2010 and grouped according to specific questions in a Microsoft Excel 2010 spreadsheet by two research assistants and co-authors, J.M.A. and S.H. All digitized answers were read and approved as being identical to the original by the author T.B. The grouped responses were subsequently analyzed, inspired by the Grounded Theory method [21], by coding each answer in Microsoft Excel 2010. Results were then compared and discussed between the authors and the main themes emerging during the coding and analyzing process were categorized. Since this was a qualitative study and due to the small, non-random sample size, no statistical analyses were carried out.

## Results

### Demographics and ART regimen

*Demographic data are presented in Table*1*and ART regimens in Table*2*.* Seventeen women and eleven men were interviewed. Residence was both urban settings in Maseru, and rural with up to 1 day’s travelling distance to the clinic. The majority of respondents had some degree of schooling (89.3 %). A wide range in household income was seen, with a substantial part of respondents reporting dependence on a family member’s income. Duration on ART varied between 2 months and 6 years with a mean of approximately 3 years, and the majority of patients were on a once daily (OD) ART regimen with one Nucleoside Analogue Reverse-Transcriptase Inhibitor (NRTI), one Nucleotide Analogue Reverse-Transcriptase Inhibitor (NtRTI) and one Non-Nucleoside Reverse-Transcriptase Inhibitor (NNRTI).

**Table 1:** Demographic data on respondents (*n* = 28)

Characteristics	Value
Age, mean	39.8 years (range 22–56 years)
Sex	17 females (60.7 %), 11 males (39.3 %)
Years of schooling, mean	6.96 years (10.7 % had no education)
Household income per month (RSA Rands)^a^	Mean: 759, Median: 600 (Range: 0–3000)
Number of people per household	Some are living together with their siblings, parents or older relatives and others have their own families with a partner and typically 1–2 children. A few patients lived on their own and the main reason was that their partner had passed away.
Treatment duration, months	Mean: 35.9, Median: 36 (Range: 2–72)
Type of treatment	OD: 19 (67.9 %), BD: 9 (32.1 %)
CD4-cell count (cells/mm^3^), mean	442 (Range: 9–1300)

^a^Mean income was calculated based on the household’s total reported income, regardless of whether the patient contributed or the number of individuals in the household. A few reported no income. Three participants were excluded, one because the patient had not responded to the question and two because of uncertainty about the amount of income

**Table 2:** ART regimen

Drugs^a^	OD	BD	*n* = 28 (%)
TDF, 3TC, EFV	12	1	13 (46.4)
TDF, 3TC, NVP	1	4	5 (17.9)
AZT, 3TC, EFV		1	1 (3.6)
AZT, 3TC, rtv/LPV		1	1 (3.6)
D4t, 3TC, EFV		1	1 (3.6)
Cannot remember the name of the drug	6	1	7 (25)
Total	19	9	28 (100)

^a^Nucleoside analog reverse-transcriptase inhibitors (NRTIs): 3TC (Lamivudine), AZT (Zidovudine), d4T (Stavudine); Nucleotide analog reverse-transcriptase inhibitors (NtRTIs): TDF (Tenofovir); Non-nucleoside reverse-transcriptase inhibitors (NNRTIs): EFV (Efavirenz), NVP (Nevirapine); Protease inhibitors (PIs): rtv/LPV (Ritonavir/Lopinavir)

### Information received during counseling

The respondents reported that they received adherence counseling when they initiated ART but no one reported explicitly having received education, counseling or advice since then. One patient reported not having received any counseling since 2004, another patient reported not having received any counseling at all and a third patient could not remember what he was counseled about because he was very ill at the time.

*“I don’t know because I was very ill. Cannot remember about how and when to take the medicine.”* 44-year old male

The message many patients recalled from their counseling was to take the medicine at the exact same time every single day, eat well and keep good hygiene, not to drink alcohol and the importance of monthly pill-refills.

*“Yes, they told me that I had to take the pill at the same time and eat well and live in a clean place.”* 54-year old female

Patients were informed that alcohol had negative effects on adherence and health. A couple of patients mentioned being advised against traditional medicine.

*“I was counseled and asked to stop taking traditional medicine because it would lower CD4 and rise viral load. Because the virus comes up I will lose weight and get other diseases.”* 24-year old female

Patients were recommended to use the “Mokoko” (“Mokoko” is the word for rooster in Sesotho), which is a 1-sheet, 1-month diary with symbols (e.g. a rooster for the morning, a sun for midday and a moon for evening), to help remember their doses. The patient can then check-off every time a dose has been taken on any given day. It was also used by the clinicians at the monthly clinic visits for adherence control.

*“They told me to mark mokoko at same time every day. Stop beer and smoke. Stop thinking stress. Exercise every day.”* 49-year old male.

It appeared that not all patients knew about their possibility to send friends or family members to refill ART at the Senkatana HIV clinic.

*“We must be allowed to send other people to pick up med’s. The service is slow…when picking of files [medical records]. There are three sick-leaves in a month sometime! Better if all together.”* 35-year old female

### Missed doses and the role of timing of doses

*Patients were asked what they would do if the time of correct dosing had been surpassed (Table***3***).* Sixteen out of 26 (61.5 %) patients (two patients did not respond) reported that they had missed one or more doses of medicine during their time on ART (mean time on ART: 35.5 months). Only seven patients responded to the specific questions about how many days they had missed their ART during the last month (mean: 4.7 days, range: 0–17) and year (mean: 15.6 days, range: 1–72).

**Table 3:** Consequences of delayed doses

Question	Answer	OD (*n* = 19)	BD (*n* = 9)
After how many hours would you skip a dose if you were late?	I would skip if I was 1 hour late	1	2
	I would skip if I was 3 hours late	2	1
	I would skip if I was 4 hours late	4	
	I would skip if I was 5 hours late	1	
	I would skip if I was 6 hours late	6	3
	I would skip if I was 8 hours late	1	
	I would skip if I was 12 hours late	1	
	I would take it even if 12 hours late	3	1
	I don’t know what to do if I am late		2

The majority of patients had been advised by clinic staff to take the medicine either at 7 A.M. or at 7 A.M. and 7 P.M, but a few was informed to choose the time of dosage on their own.

*“How and when [at the pharmacy] to take med’s, choose time myself, every day, not wait with refill until finished.”* 36-year old male

*“Stick to the chosen time and be punctual.”* 42-year old female

One patient explained that she had been told by a clinician to skip her daily dose, if she was more than 30 minutes late.

*“Take them daily at the same hour, not after 30 minutes. Take them for the rest of life.”* 29-year old female

Several patients admitted that they would skip doses of ART if they came in a situation of being just a few hours late (*Table***3**) and two patients had skipped doses of ART in the past because they were late.

*“I stay with my nephews and when they fight I forget to take the pills. They fight with fists. They were in the bedroom, I was in the kitchen. I keep the pills in the bedroom. I am not afraid but forget. I remember later but was told [by clinic staff] to skip [ART] if too late.”* 34-year old female

*“Leave it, even if only 1 hour late, and ask counselor. It happened once when my wife passed away, never again.”* 47-year old male

### Barriers to adherence

*Barriers to ART-adherence reported by two or more respondents are shown in Table*4. The reason most frequently listed by the patients as a barrier to adherence was a **change in routines or an unpredicted event**, which could cause the patient to miss doses for various time periods, ranging from days to weeks.

**Table 4:** Barriers to ART-adherence

Answer^a^	*n* = 28^b^
Change in routine or an unpredicted event	10
Not bringing pills along when leaving the house	6
Running out of pills or being prevented from picking up refills	5
Alcohol	4
Work	4
Water and/or food were not available	3
Skipping doses of ART as told by clinic staff when he/she was late	2

^a^Barriers mentioned by two respondents or more were included in the table

^b^Respondents were allowed to mention as many options as preferred. Thus the number of response categories per person to each question could be higher than one

*“The cows were stolen and I took a week looking for them and had forgotten the medicine. 15 cows in the mountains were missing – I walked to get them.”* 42-year old male

*“I was stressed because attackers wanted to kill my son at my home, and I forgot to pick up the medicine. I was forgetting everything even to eat for at least 3 weeks. Others have already been killed at night with guns, and animals are stolen.”* 47-year old female

Similarly, one woman said she attended a funeral, another had to take a sick child to the hospital, and a man was prevented from leaving his job because of a flood that made medicine unavailable at dose time.

The issue of **not bringing pills along when leaving the house** was mentioned as a reason for having missed one or more doses of medicine.

*“I was in a hurry to work. The past 3 months I have been working in a factory, I get up at five and meet at seven. I carry [ART] in my pocket, but sometimes I forget to bring it along with me.”* 32-year old male

*“I can forget it, if I am busy at work. It happens sometimes. I am a farmer, and in the morning and in evening the doses can be missed. I work 2 hours away. I bring the tablets along if I know I will be busy… but sometimes I did not know I would be busy, or I was travelling, and there was no water. I cannot take it without water.”* 52-year old male

*“I am a driver. They can ask me to go to RSA or other countries and not sleep at home. Sometimes I could not take [ART].”* 43-year old male

Another problem was **running out of pills or being prevented from picking up refills**. In some cases the reason for why a patient had not managed to refill in time was simply forgetfulness. Others mentioned being out of town or unable to pick up refills. In the following example the patient arrived late at the clinic and could not refill her pill bottle because the clinic was closed for the day.

*“This last Friday I came after 12 [A.M.] and the reception was closed. The people were there but did not want to unlock. I was late because I had problems with the taxi. I have been told to be here at 11, but the taxi broke on the way. I missed [taking ART] Sunday through Monday and Tuesday morning.”* 46-year old female

**Alcohol** was a barrier mentioned by a few patients. One patient explained that he would skip his medicine when he had been drinking due to concerns about interactions with alcohol. Another patient missed several doses due to forgetfulness caused by alcohol.

*“Yes, I was drinking beer, and was forgetting an entire week. Then I was taken to the counselor by the grandmother. It happened for only 1 week*.” 25-year old male

**Work** (meetings, long and busy working days or advice to take ART during working hours) would cause patients to postpone or miss one or more doses of ART.

*“Once I postponed for three hours because I was in a meeting.” 42-year old female*

Another barrier to adherence was that **water and/or food were not available** when it was time to take the medicine. Some patients felt that the medicine made them hungry and when food was not available they would skip a dose. Another patient would not be able to swallow the pills without water.

*“I was not forgetting but I defaulted because I had nothing to eat, and pills make me hungrier.”* 33-year old female

*“I missed a dose of medicine 2 weeks ago. I was on a journey, and I had no water, but I had the tablets in my pocket.”* 52-year old male

Two patients reported **skipping doses of ART as told by clinic staff when he/she was late** with a dose. (See citations in *Missed Doses and the role of Timing of Doses.)*

A few reported that they missed daily doses of medicine because they **simply forgot** or **slept over** and one patient explained that the night-dose in a bi-daily (BD) ART regimen was the most difficult to remember.

### Facilitators of adherence

*The facilitators of ART-adherence reported by two or more respondents are shown in Table***5***.* A large number of respondents listed the **mobile phone alarm and/or mobile phone clock** as the primary tool to help remember taking ART. Not all patients had a mobile phone of their own, but most households had access to one. The mobile phone often belonged to a younger family member.

**Table 5:** Facilitators of ART-adherence

Answer^a^	*n* = 28^b^
Mobile phone alarm and/or mobile phone clock	18
Support from family and/or friends	17
TV and Radio	12
Regular clock or alarm clock	9
HIV-positive partner, relative or friend	7
Remember the medication pontaneously	5
Previous severe symptoms of HIV infection	3
Mokoko^c^	2
Planning ahead	2
Disclosure of HIV serostatus	2

^a^Facilitators mentioned by two respondents or more were included in the table

^b^Respondents were allowed to state/mention as many options as preferred. Thus the number of response categories per person to each question could be higher than one

^c^Mokoko (meaning “rooster” in Sesotho), is a markable 1-sheet 1-month calendar built up by symbols (e.g. A rooster for the morning, a sun for the day and a moon for the evening) to help patients remember their doses

*“I am reminded by my husband and by our phone alarm. The alarm rings at seven when I take it, and eight, when he takes it.”* 36-year old female

*“My children say:” Daddy it is time to take the medicine” when they hear the phone alarm.”* 43-year old male

**Support from family and/or friends** was a frequently reported facilitator of ART-adherence.

*“My sister reminds me, we live together, she is HIV-unknown, but looks sick. Puts them [the pills] where I can see them.”* 33-year old female

*“My children. They made a song called Sogum [the medicine] – “Mummy it is time for Sogum”.”* 45-year old female

*“Wife or children [remind him] if phone battery is charged far away from home.”* 44-year old male

**TV and radio** or using a **regular clock or alarm clock** also played a dominant role in helping patients remember to take the medicine on time.

*“Radio. I am a babysitter, listens all the time.”* 39-year old female

*“Radio. I do not know how to read the clock.”* 32-year old female

*“Watch, no alarm, or TV. I have no cell phone.”* 22-year old female

*“Looks at the clock on the wall. If not home – I have another watch. Have no cell phone. Takes it after meal or if no food just takes them.”* 56-year old female

Another facilitator of adherence was to have an **HIV-positive partner, relative or friend** to share the experience with or get reminders and support from.

*“My wife reminds me, she is also on ARV’s, also takes them seven, but BD.”* 41-year old male

*“My neighbor, who is also positive – asks: “now it is 7 o’clock, did you take it?”.”* 39-years old female

Several patients stated that they would **remember their medication spontaneously.** Three patients mentioned **previously severe symptoms of HIV infection**, which motivated them to stay adherent to ART. After initiating ART one patient began to feel better and explained:

*“I have the interest in taking them [the pills] because I was very sick and got better after taking them.”* 47-year old female

Two patients used the **mokoko** to remember their daily doses of ART and two other patients were **planning ahead**, typically by bringing ART along when leaving the house. For example a 36-year old male always made sure he was home when it was time to take the 7 P.M. dose and he would bring ART along on travels.

Two respondents reported predominantly positive emotions and feed-back when they **disclosed their HIV serostatus** to family members and co-workers.

*“It helps that I have disclosed to boss and all co-workers.”* 32-year old male

## Discussion

In this qualitative study we discovered “surpassed medication intake time” as an issue, and mapped obstacles and facilitators of adherence to treatment. A change in routine, an unpredicted event or leaving home without pills was often mentioned as obstacles to ART-adherence. Use of mobile phones and social support appear to be key factors in ensuring adherence to ART.

The non-randomly selected group of adult respondents had a majority of women and an education level slightly higher than the overall estimated mean years of schooling in Lesotho, (6.9 vs. 5.9 years (2012)) [22], in congruence with the higher HIV prevalence in women [23], the slightly higher prevalence of women in Lesotho and the urban–rural disparities in school attendance [24].

The interviews indicated that the patients did not receive adherence counseling as frequently as would be optimal. It is known that adherence is not static, but is a dynamic process and tends to decline over time [15–17]. Increasing the frequency of education and counseling could therefore be an area of intervention to improve adherence in this setting, as shown in earlier Sub-Saharan studies [25].

We found that the respondents were generally told by the counselors to take their medication “on time” and unfortunately, several patients were willing to skip their medication, if they discovered they were late by just a few hours. Two patients were **skipping doses of ART as told by clinic staff when he/she was late**. This iatrogenic barrier has to our knowledge not been explored elsewhere, although it has been reported as a barrier to ART adherence by a health care provider and three patients who were failing second-line ART in a small study from RSA [26]. In more affluent countries, forgetfulness has shown to be an important barrier to ART adherence and it has been suggested to advice patients to link the daily dosage with a daily routine such as tooth brushing or having breakfast [20]. However, patients reported that inconsistency in daily routines was a primary barrier to adherence. Therefore, coupling medication intake with daily routines may be sub-optimal in this setting. There are no large randomized controlled trials which compare regimens of “on time” with “routine coupled” dosage of ART in Sub-Saharan Africa. In a small pilot study viral suppression was maintained at 24 and 48 weeks on intermittent treatment with an Efavirenz-based regimen (5 days on and 2 days off) [27]. In contrast, studies of longer (≥1 week) ART interruptions have resulted in increased viral load [28, 29]. We suggest that physicians should emphasize to patients the importance of taking a delayed dose as soon as they remember, instead of skipping the dose completely.

The main barriers to adherence in this study were related to hassles of daily life, such as a **change in routine or an unpredicted event. Not bringing pills along when leaving the house** was also a frequently mentioned barrier. Other researchers have reported being away from medicine at dose time as the main reasons for non-adherence [30]. Patients should be advised to maintain a travel kit filled with sufficient medication for the duration of their absence from home. Pill burden was not reported as a barrier to ART adherence in this setting but one patient found the night dose in a BD regimen as more difficult to remember than the morning dose. The majority of patients were on a OD ART regimen which could improve adherence as opposed to BD regimens [31].

A few patients had skipped doses of ART because **water and/or food were not available** at dose time. Lack of food [12, 14] and hunger [30, 32–34] have previously been reported as barriers to adherence to ART in sub-Saharan Africa. Cantrell et al. conducted a pilot study in Zambia, showing improved adherence with food supplements delivered to HIV patients on ART [35]. Other solutions could be to advice patients to always carry a bottle of water and a snack together with the daily ART. **Alcohol** is a problem in Sub-Saharan Africa, especially with its unfortunate effects on HIV transmission rates and ART adherence [36]. In our sample of patients, alcohol was reported as a barrier to ART adherence by four patients. The majority of patients recalled being counselled not to drink alcohol. Some patients **were running out of pills or being prevented from picking up refills**. At the Senkatana HIV clinic in Lesotho it is an option to send friends or family members to pick up ART at the clinic, but this was not known to all of the respondents.

In this study the **mobile phone alarm and/or mobile phone clock** was often used by patients or their friends and family as medication reminders. A recent qualitative study from Ethiopia report the same use of mobile phones as a reminder tool [37]. Other studies have found positive evidence with supportive text messages and other reminder devices [25, 38, 39]. At the clinic in Maseru, reminding patients by sending text messages was not an option at the time of the study. With an estimated 86.3 mobile-phone subscriptions per 100 inhabitants in Lesotho in 2013 [40] and the growing number of mobile phones in Africa [41–43], this practice could face widespread application. Most respondents had access to a mobile phone in this setting and thus, using Short Message Service in ART-clinics could be a future option. **Support from family and/or friends** facilitated adherence, for example the children of the person taking ART. The literature suggests school education programs for children to strengthen their role as treatment supporters [38]. Recommending patients to bring family members along to counseling and to treat family members together has shown to have a positive impact on adherence [15]. We saw a tendency to form alliances with an **HIV-positive partner, relative or friend** and peer-counseling has been shown to have a positive effect on adherence [44]. Several patients in this setting used the **TV or radio** as a reminder and other studies confirm this method’s frequent use [38]. We suggest that clinicians discuss with the patient the preferred facilitators, and encourage its use.

One of the major obstacles to improvement of HIV/AIDS health care in Sub-Saharan Africa is to ensure that all patients eligible for ART have access to ART. In 2012 an estimated 59 % of all eligible patients received ART in Lesotho [1]. Once ART is initiated an estimated 72 % of the patients in Lesotho are still on ART 12 months after commencement [1]. Thus retention to ART and accessibility are important issues to address. Addressing these issues may have a greater impact on survival than simply focusing on individual adherence.

To address these major challenges we suggest: introducing more flexible refill-practices, telling patients that family members are allowed to pick up medicine, introducing rations that last for more than a month, group sessions to encourage peer-support, more frequent counseling including education about disease and treatment, and sending text messages in advance to remind patients that it is time for a refill. Actions costing money like additional counseling sessions or text message services may not be realistic to implement in HIV-clinics of recourse limited countries. However, the existing counseling may be improved concerning the information given. Adherence and quality of life may be improved by allowing the patients to be more flexible with regard to timing of doses, for instance by taking the medicine in combination with a daily routine or to take the dose as soon as they remember instead of skipping it completely if they are delayed, and informing the patients about the potentials of mobile phones and support from family and friends. Specifically, counselors could focus on the scenario of an upcoming unexpected event or a change in routines and how to prepare patients for these situations. Finally we believe that it is of great importance that counselors are attentive towards patients’ preferred methods of remembering their ART doses and try to facilitate their good behavior.

### Strengths and limitations

A strength of this study is that we explored in depth the barriers and facilitators, and that the issue of “surpassed medication intake time” was discovered. The in-depth interviews in native language enhance trustworthiness of the findings. However, there are also some limitations to our study. The relatively few respondents, the fact the researchers did not speak the local language, and the lack of taped interviews. Another limitation to our study is that only patients were interviewed in depth. It would have been optimal to be able to compare patients’ and clinicians’ point of views. Finally, the findings of this study cannot necessarily be generalized to other countries or parts of Lesotho due to the small, non-randomly design. However, the issues of timing of doses of ART and the widespread use of mobile phones may well be useful in other Sub-Saharan African HIV clinics. This study confirms the complexity of barriers to and facilitators of adherence to ART.

## Conclusions

Our findings suggest that patients are often told by clinicians to be strictly on time with their doses of ART and unfortunately several patients would skip a dose if they came in a situation of being only a few hours late. Patients should be advised to take their medicine immediately when they remember, instead of skipping it altogether when late. We hypothesize, that in some settings it may be easier for patients to be adherent to ART if the medication is linked to daily routines, for example tooth-brushing, but the literature lacks large randomized clinical trials comparing medication intake at exactly the same time each day with medication intake linked to daily routines. A major barrier to ART adherence was interruptions of daily routines and therefore coupling medication intake to daily routines may be unsuitable in this setting. Leaving the house without sufficient medicine was also listed as an important barrier and patients should be advised to maintain a travel kit. Mobile phone alarms and family support were major facilitators of adherence and is supported in the literature. None of the patients reported to have been counseled on family support or the use of mobile phones as helpful methods to improve adherence to ART. These areas could be subjects of focus during counseling particularly with the growing numbers of mobile phones in Africa and the current focus on telemedicine.

## Abbreviations

ART: Antiretroviral therapy
BD: Bi-daily
HIV: Human immunodeficiency virus
NRTI: Nucleoside analog reverse-transcriptase inhibitor
NtRTI: Nucleotide analog reverse-transcriptase inhibitor
NNRTI: Non-nucleoside reverse-transcriptase inhibitor
OD: Once daily
PI: Protease inhibitor
PLWH: People living with HIV
RSA: Republic of South Africa
